# Comparative genome analysis of *Mycoplasma pneumoniae*

**DOI:** 10.1186/s12864-015-1801-0

**Published:** 2015-08-16

**Authors:** Li Xiao, Travis Ptacek, John D. Osborne, Donna M. Crabb, Warren L. Simmons, Elliot J. Lefkowitz, Ken B. Waites, T. Prescott Atkinson, Kevin Dybvig

**Affiliations:** Department of Medicine, The University of Alabama at Birmingham, Birmingham, AL 35294 USA; Department of Microbiology, The University of Alabama at Birmingham, Birmingham, AL 35294 USA; Department of Pathology, The University of Alabama at Birmingham, Birmingham, AL 35294 USA; Department of Genetics, The University of Alabama at Birmingham, Birmingham, AL 35294 USA; Department of Pediatrics, The University of Alabama at Birmingham, Birmingham, AL 35294 USA; Center for Clinical and Translational Science, The University of Alabama at Birmingham, Birmingham, AL 35294 USA

**Keywords:** Arginine deiminase, CARDS toxin, Epigenetics

## Abstract

**Background:**

*Mycoplasma pneumoniae* is a common pathogen that causes upper and lower respiratory tract infections in people of all ages, responsible for up to 40 % of community-acquired pneumonias. It also causes a wide array of extrapulmonary infections and autoimmune phenomena. Phylogenetic studies of the organism have been generally restricted to specific genes or regions of the genome, because whole genome sequencing has been completed for only 4 strains. To better understand the physiology and pathogenicity of this important human pathogen, we performed comparative genomic analysis of 15 strains of *M. pneumoniae* that were isolated between the 1940s to 2009 from respiratory specimens and cerebrospinal fluid originating from the USA, China and England.

**Results:**

Illumina MiSeq whole genome sequencing was performed on the 15 strains and all genome sequences were completed. Results from the comparative genomic analysis indicate that although about 1500 SNP and indel variants exist between type1 and type 2 strains, there is an overall high degree of sequence similarity among the strains (>99 % identical to each other). Within the two subtypes, conservation of most genes, including the CARDS toxin gene and arginine deiminase genes, was observed. The major variation occurs in the P1 and ORF6 genes associated with the adhesin complex. Multiple *hsdS* genes (encodes S subunit of type I restriction enzyme) with variable tandem repeat copy numbers were found in all 15 genomes.

**Conclusions:**

These data indicate that despite conclusions drawn from 16S rRNA sequences suggesting rapid evolution, the *M. pneumoniae* genome is extraordinarily stable over time and geographic distance across the globe with a striking lack of evidence of horizontal gene transfer.

**Electronic supplementary material:**

The online version of this article (doi:10.1186/s12864-015-1801-0) contains supplementary material, which is available to authorized users.

## Background

*Mycoplasma pneumoniae* is a parasitic bacterium belonging to *Mollicutes,* a class of bacteria lacking cell walls and typically having small genomes under 1,000 kb. It is a common pathogen of the upper and lower respiratory tract of humans in all age groups worldwide. It is also the most comprehensively analyzed species of *Mycoplasma*, with recent studies characterizing *M. pneumoniae*’s transcriptome, proteome and metabolome [1–3]. *M. pneumoniae* causes up to 40 % of community acquired pneumonias [4]. Although the infection is mild in most cases, patients can occasionally develop severe to fatal diseases. In addition to respiratory infections, as many as 25 % of *M. pneumoniae* infections are accompanied by extrapulmonary complications, which can affect almost any organ system either by direct infection or by infection-associated autoimmune phenomena [4, 5].

The pathogenicity of *M. pneumoniae* is still under active investigation and several virulence mechanisms have been identified. *M. pneumoniae* is primarily an extracellular pathogen requiring close association with host cells to survive as its highly reduced genome renders it incapable of *de novo* synthesis of amino acids, nucleotides, and other essential molecules. Also, the mycoplasmas are unique among bacteria in their growth requirement for host cholesterol. Adherence to the host respiratory epithelium is believed to be the initiating event that facilities local cell injury, tissue disruption, and cytotoxic effects [4]. Several protein components of the adhesin complex have been identified including the P1 protein. The two *M. pneumoniae* subtypes, type 1 and type 2, were established based on P1 sequence polymorphisms [6]. Hydrogen peroxide and superoxide radicals are known virulence factors of *M. pneumoniae* [7, 8]. The Community Acquired Respiratory Distress Syndrome (CARDS) toxin, an ADP-ribosylating and vacuolating toxin of *M. pneumoniae*, is capable of inducing pulmonary inflammation and airway hyperreactivity [9–14]. Inappropriate host immune responses also contribute to the pathogenesis of *M. pneumoniae* infection. The molecular mimicry by *M. pneumoniae* adhesin proteins and glycolipids of various host cell components may trigger autoimmune disorders that involve multiple organ systems [4, 15]. *M. pneumoniae* may also be a facultative intracellular pathogen; viable bacteria have been shown to move into the interior of human cells *in vitro* [16]. This aspect of the organism’s life cycle and the ability to form biofilms on epithelial tissue likely contribute to the establishment of chronic infection [17].

Whole genome sequencing has greatly facilitated our understanding of *M. pneumoniae*. At present there are 4 distinct *M. pneumoniae* strains completely sequenced. The genome of the type 1 strain M129 (ATCC 29342) was sequenced by using a laborious approach involving the construction of an ordered cosmid library. The sequence was reported in 1996 and reannotated in 2000 as having 816,394 bp, 730 genes, and an average GC content of 40 % [14, 18]. The genome sequences of the type 2 strains FH and 309 were completed using next generation sequencing methods (Roche 454 sequencers) [19, 20]. Another strain, M29 was recently submitted (accession number GCA_00733995.1) and has not yet been annotated. A preliminary comparison of the first three genomes indicated that they are very similar, except for variation in a 6-kb insertion region coding lipoproteins [20]. We have resequenced M129 and FH and also have sequenced 13 additional strains obtained from different geographic regions over a period of several decades. For each strain, the sequence was completed to generate a single, circular contig. Analysis of the genomes reveals numerous differences between type 1 and type 2 isolates but a striking degree of homogeneity between strains of the same type, suggesting clonality.

## Methods

### *M. pneumoniae* strains

A total of 15 *M. pneumoniae* strains were sequenced in this study (Table 1), including 11 clinical isolates and 4 reference ATCC strains. These strains were originally isolated over a wide period of time, 1944 to 2009, and geographic range, North America, Asia and Europe. Except for one specimen from cerebrospinal fluid, most of these clinical strains were isolated from respiratory specimens after minimal growth in culture media. There is one macrolide resistant strain from the US (54089). The ATCC strains were purchased from ATCC in 2002, grown in 2005, and the 3^rd^ passage was used in this study.

**Table 1:** Summary of the sequenced strains

Strain		Description	Isolation source	Date collected	Site originated
Type 1	M129	ATCC 29342 (Reference Strain)	Patient with pneumonia	1968	USA/NC
	142.8	ATCC 29085	Throat	1960	USA/MD
	51494	Clinical isolate	Cerebral spinal fluid	2006	USA/CO
	54089	Macrolide resistant isolate	Throat	2009	USA/AL
	54524	Low passage isolate	Throat	2009	USA/AL
	85084		Respiratory specimen	Prior to 1985	China
	85138		Respiratory specimen	Prior to 1985	China
Type 2	FH	ATCC 15531 (Reference Strain)	Sputum	1954	USA/MA
	19294	Low passaged isolate	Throat	1994	USA/OH
	39443	Low passaged isolate	Throat	1999	USA/AL
	M1139		Respiratory specimen	1981	England
	M2192		Respiratory specimen	1982	England
	M2592		Respiratory specimen	1982	England
	MAC	ATCC 15492	Human lung tissue	1944	USA/CA
	UAB PO1	Low passage isolate	Throat	1980	USA/AL

### Culture and DNA preparation

All strains were grown in 25 ml SP4 medium in T-flasks at 36.5 °C until color change. Non-adherent organisms were discarded. The adherent organisms were gently washed twice with 10 ml PBS (phosphate buffered saline, pH 7.4), scraped from the bottom of the flasks, and suspended in 5 ml PBS. Genomic DNA was purified using the QIAamp DNA Blood Maxi Kit (Qiagen, Valencia, CA) according to manufacturer’s instruction. After determining the DNA concentration (NanoDrop 1000, Wilmington, DE) and quality (0.8 % agarose gel), all DNA products were stored at −80 °C until use.

### Next-generation sequencing (NGS)

NGS of all *M. pneumoniae* strains was performed using the Illumina MiSeq platform in the UAB Heflin Genomic Core. Paired-end 250-bp reads were used.

### Genome assembly and annotation

NGS sequencing reads were assembled *de novo* using ABySS v 1.3.7 [21]. Kmer values were tested iteratively to find the value yielding an assembly with the lowest number of contigs, while retaining a total contig length of approximately 800 kb. The s parameter was changed to double the kmer value, per recommendations by the developer. All other parameters were set to default. The number of contigs generated ranged from 5 to 13 per strain were generated by *de novo* assembly. These contigs were mapped to the M129 reference genome using BLAT [22] and visualized using IGV [23, 24]. This mapping was used to develop PCR primers to join the contigs. High fidelity PCR reactions and Sanger sequencing were performed using standard methods. Overlapping and joining of the contigs was performed manually with the aid of HVDR fragment merger tool [25] and Audrius Meskauskas’s reverse complement tool [26]. Completed, circularized genomes were annotated using RAST [27, 28], the NCBI prokaryotic pipeline [29] and manual reconciliation.

### Single nucleotide polymorphism (SNP) and insertion/deletion (indel) analysis

To call SNPs and indels, completed genomes were first broken into 10-kb “reads” at 1-kb intervals and then aligned to either the M129 or FH reference strains (NCBI accession numbers NC_000912 and NC_017504, respectively) using BWA v0.7.7 [30]. The resulting BAM files were used as input for GATK v3.0-0 [31]. We used GATK’s Unified Genotyper and Haplotype Caller to call SNPs and indels. Because the “reads” came from an assembled genome, a perfect quality score was assigned to each base. This necessitated the use of the allow PotentiallyMisencodedQuals parameter when running GATK’s Realigner Target Creator. Otherwise, GATK was run using standard parameters according to GATK Best Practices recommendations [32, 33]. The effects of the SNPs and indels in the resulting VCF files were evaluated using snpEff v3.3 [34]. Although snpEff does annotate each SNP and indel with the gene that they fall into, we reannotated the VCF files using the latest annotations of the M129 and FH genomes downloaded from NCBI.

### Functional annotation

Functional annotation of gene lists was performed using BRITE search from the Kyoto Encyclopedia of Genes and Genomes (KEGG) [35].

### Protein sequence analysis

Gene sequences were downloaded from the RAST server after annotation. To identify specific genes, these sequences in FASTA format were compared to the reference sequence as found on NCBI by using BLAST. Genes were translated using the translation tool at Bioinformatics Organization [36], and the protein sequences aligned using CLUSTAL Omega [36].

### Comparative genomics

Completed genomes were aligned using BRIG [37] to visualize overall sequence similarity between the strains. The annotated genomes, in the form of GenBank files from RAST, were aligned with MAUVE [38] to identify structural variations and which genes they may affect. For phylogenetic tree generation, completed genomes were aligned using MAFFT [39, 40] via the CIPRES science gateway [41]. We generated phylogenetic trees from the genome alignment using MrBayes [42]. To generate trees for protein sequences, Clustal X [43] was used to align protein sequences and to generated trees. For both genome sequence and protein sequence trees, 1000 iterations of boostraping analysis were used to generate confidence values. Trees were visualized using Dendroscope [44, 45] and FigTree [46]. Tandem repeats across the genome were identified by Tandem Repeat Finder 9 [47].

## Results

### Genome assembly

We sequenced the 15 *M. pneumoniae* strains with NGS and computationally *de novo* assembled them into contigs. The characteristics of these assemblies are found in Additional file 1: Table S1. The resulting contigs were mapped to the M129 reference genome and joined via PCR. All fifteen genomes had all contigs joined to form a single, continuous (circular) contig. Following assembly and editing, the genomes underwent automated gene annotation. Summary statistics for the completed genomes, including submission numbers are found in Table 2. These genomes, having about 40 % of GC and ranging from 816402 to 818633 bp, code for a total of 853 to 870 genes.

**Table 2:** Characteristics of the completed genome assemblies

Strain		Accession	Length	%GC	Genes			
					CDS	rRNA	tRNA	Total
Type 1	M129	CP003913	816451	0.40038	790	6	74	870
	142.8	CP010538	816496	0.40011	790	6	74	870
	51494	CP010541	816404	0.40005	781	6	74	861
	54089	CP010542	816565	0.40010	784	6	74	864
	54524	CP010543	816583	0.40009	780	6	74	860
	85084	CP010544	816404	0.40011	788	6	74	868
	85138	CP010545	816402	0.40011	788	6	74	868
Type 2	FH	CP010546	817207	0.39981	786	6	72	864
	19294	CP010539	818633	0.40001	780	6	72	858
	39443	CP010540	817184	0.39979	782	6	72	860
	M1139	CP010547	817045	0.39977	789	6	72	867
	M2192	CP010548	817169	0.39978	783	6	72	861
	M2592	CP010549	817198	0.39981	783	6	72	861
	MAC	CP010550	817156	0.39983	788	6	72	866
	PO1	CP010551	817216	0.39982	788	6	72	866

### Genome comparison

**Fig. 1:**
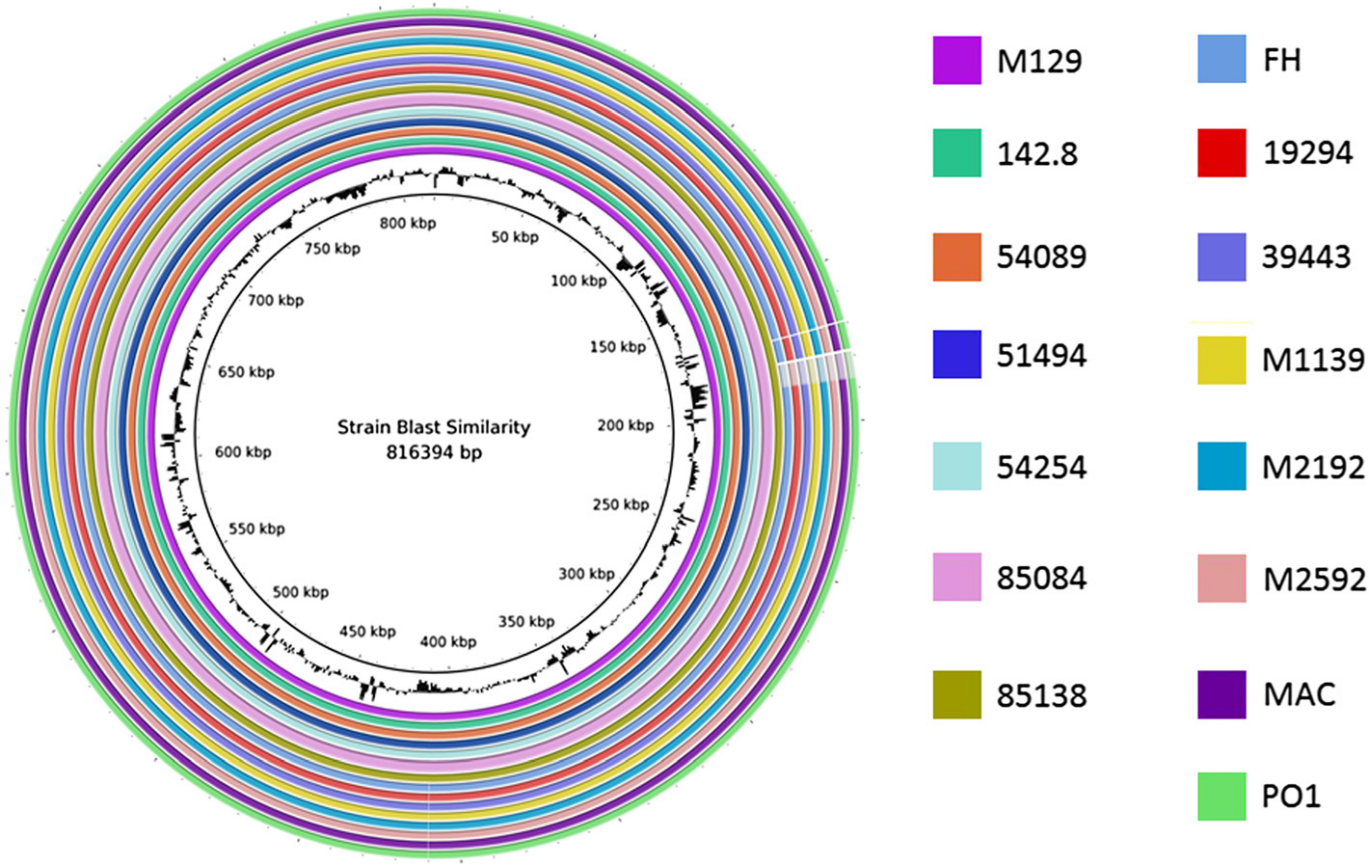
Overall sequence identity of the 15 sequenced strains with the reference M129 genome. BLAST-based similarity of a given strain versus the M129 reference is represented as a colored ring. Colors by strain are indicated to the right. Solid coloration indicates >99 % identity and transparent grey indicates approximately 95 % identity. Location in the reference genome is indicated by numeration on the inside of the ring. GC content in the reference genome is indicated by the black bar graphs between the genomic coordinates and the colored rings (bars pointing toward the outside of the circle indicate high GC content). Note that genomic structural alterations are not visible using this method

**Fig. 2:**
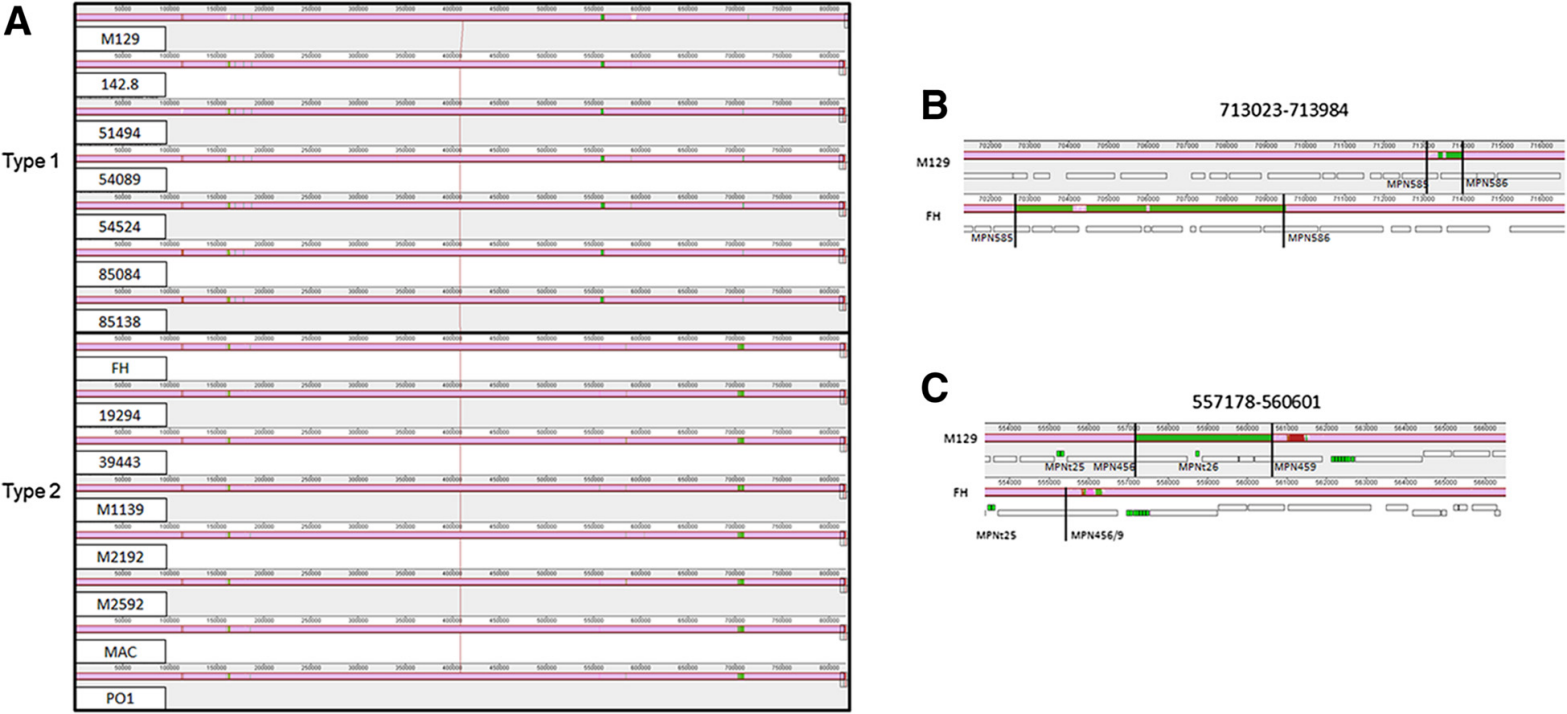
Whole genome alignment of the 15 sequenced strains using MAUVE. Regions colored in mauve are conserved across all strains. Differently colored blocks are conserved in some strains. Blocks that are lower are inverted relative to the other strains. Open boxes indicate the location of genes. tRNA genes are shaded in green and rRNA genes are shaded in red. Genes affected by the indicated variants are labeled. Numbers above intervals indicate locations relative to the M129 strain. **a** Alignment showing all 15 strains. **b** Close up of the type 2-specific insertion. M129 and FH are shown and are typical of the other type 1 and 2 strains, respectively. Lines indicate relative point of insertion. **c** Close up of the type 1-specific insertion. M129 and FH are shown and are typical of the other type 1 and 2 strains, respectively. Lines indicate relative point of insertion

**Fig. 3:**
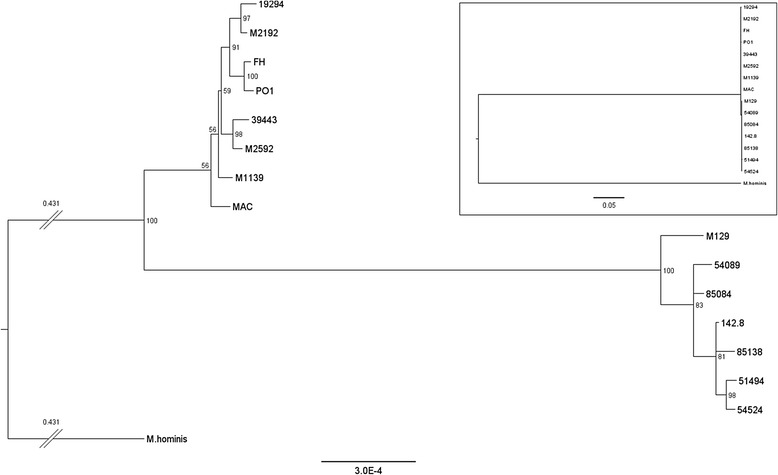
Phylogenetic tree based on whole genome alignment of the 15 sequenced strains. The 15 sequenced *M. pneumoniae* strains and *M. hominis* (included as an outgroup) were aligned, and a tree was generated using the alignment. Confidence values, represented as percent of supporting bootstrapping iterations are shown for each node. Scale, in differences per site, is indicated at the bottom. The branches between *M. hominis* and the *M. pneumoniae* strains have been truncated (indicated by double slashes), and the branch length (in differences per site) is indicated above the branch. The inset shows the same tree (rescaled, note the new scale bar) without any branches truncated

The 15 sequenced genomes were aligned using a variety of methods. To determine their overall similarity, the genomes were aligned to the reference M129 genome using BRIG, a BLAST-based alignment method. Overall, the genomes were 99 % to > 99 % identical; the similarity within each subtype group was less than 0.1 % difference among the strains. There was one distinct region where identity dropped to about 95 % in the type 2 strains (Fig. 1). This region corresponds to the P1 gene (Fig. 1). We also aligned the genomes using MAUVE to detect large chromosomal rearrangements, deletions, and duplications (Fig. 2a). MAUVE detected two subtype-specific insertions (Fig. 2b and c): the type 1-specific 557178–560601 (M129 numbering) insertion and type 2-specific 713023–713984 (M129 numbering) insertion. All of the genes affected by these insertions were hypothetical proteins, with the exception of a tRNA gene (MPNt26) in the type 1 specific insertion. MPNt26 codes for a serine TCG tRNA. Both type 1 and type 2 strains have another serine TCG tRNA gene (MPNt25) immediately upstream of the insertion point. The genomes (including *M. hominis* as an outgroup) were aligned with MAFFT and a phylogenetic tree was generated using MrBayes (Fig. 3). Not surprisingly, the 15 strains fall into 2 groups representing type 1 and type 2.

### SNP and indel analysis

SNPs and indels were compared relative to type 1 and type 2 reference strains, M129 and FH, respectively. To our knowledge, there is no whole-genome alignment program that generates an alignment file that can be used with current SNP and indel callers like GATK. Instead of going back to the un-assembled short reads, we broke the completed genomes into 10-kb “reads” in 1-kb intervals, aligned these “reads” to the genome, and used the resulting BAM file as input for GATK. We tested GATK’s UnifiedGenotyper and HaplotypeCaller for genotyping. HaplotypeCaller is newer and calls indels better, but lacks a haploid-genome setting. Overall, the results were highly similar for the two SNP callers. For a subset of genes that were examined, SNP and indel calls were almost perfectly concordant. For indels involving tandem polynucleotide repeats, UnifiedGenotyper missed a few indels found by HaplotypeCaller. We therefore utilized the results from HaplotypeCaller. SNP and indel effects were annotated with snpEff, and we manually re-annotated gene symbols for the SNPs using the latest gene annotations of the M129 and FH reference strains from NCBI.

The SNP and indel counts, by predicted functional effect as annotated by snpEff, relative to the M129 (type 1) reference genome are found in Table 3. As expected, type 1 strains showed fewer variants (235-431) than type 2 strains (1569–1615). As a test of the system, the M129 reference sequence downloaded from NCBI was processed in the same way as the 15 sequenced strains and had SNPs called against itself. No variants were found. SNPs and indels were also called against the FH (type 2) reference genome, and the results were similar with about 1500 variants and 200 variants detected in type 1 and type 2 strains, respectively. No variants were detected when running the FH reference genome against itself. However, the gene annotation for the M129 reference genome appeared to be more complete with fewer unnamed genes. Therefore, all further variant analysis was done using the M129 (type 1) reference genome.

**Table 3:** Variants relative to the M129 reference strain

Strain		Total variants	Non-synonymous genic SNPs				Synonymous genic SNPs	Genic indels		IntragenicSNPs and indels
			Coding	Start lost	Stop gain	Stop lost		In frame	Frameshift	
Type 1	M129	235	113	0	3	0	41	10	36	32
	142.8	334	174	1	2	1	67	4	22	63
	51494	382	202	1	3	1	77	7	24	67
	54089	431	223	1	3	1	90	8	36	69
	54524	385	208	1	2	1	76	5	22	70
	85084	404	199	1	2	1	88	8	38	67
	85138	365	183	1	1	1	72	2	25	80
Type 2	FH	1581	705	0	8	6	474	35	109	244
	19294	1606	705	0	11	6	490	33	110	251
	39443	1615	713	0	10	6	489	36	110	251
	M1139	1581	687	0	8	6	485	33	114	248
	M2192	1604	705	0	11	6	490	35	109	248
	M2592	1588	697	0	8	6	484	33	111	249
	MAC	1569	677	0	8	7	477	34	114	252
	PO1	1588	700	0	8	6	482	34	116	242

To test the accuracy of our assemblies, we compared the variants in the resequenced M129 strain relative to the original M129 reference sequence. Of the 203 genic SNPs and indels in the resequenced M129, 101 were found in all other sequenced strains or in all of the sequenced type 1 strains, suggesting that these variants were actually errors in the original M129 sequence. The remaining 102 variants represent about 0.01 % of M129’s genomic sequence. A further 56 were found in at least one other type 1 strain (in almost all cases, the variant was found in all type 2 strains or in type 1 strains 54089 and 85084). Another 10 were found in several type 2 strains, but no type 1 strains. This left 36 variants that are unique to the resequenced M129 strain. These 36 variants, the most likely candidates for sequencing errors, variant miss-calls, or new mutations, represent about 0.004 % of M129’s genomic sequence. Two of these variants were found in the MPN413 gene and the rest were found in MPN489. These two genes code for proteins of unknown function.

To explore the variable and invariable regions of the *M. pneumoniae* genome, we identified the genes with the most and least non-synonymous variants in type 2 strains compared to the M129 (type 1) reference genome. The top 10 genes with the most non-synonymous variants are found in Table 4. When looking for genes with the fewest variants, we found 182 genes with no variants in any of the sequenced strains. The list of these genes is found in Additional file 2: Table S2. For those genes that could be classified by KEGG, a summary of the functional groups into which these genes fall is found in Table 5.

**Table 4:** Type 2 genes with the most variants as compared to M129 (type 1)

Gene	Function	FH	19294	39443	M1139	M2192	M2592	MAC	PO1
MPN457	Unknown	165	164	165	165	164	165	165	165
MPN141	P1	52	67	68	65	66	67	67	66
MPN142	ORF6	17	16	16	16	16	16	16	16
MPN286	Unknown	13	14	13	13	14	13	13	13
MPN205	Unknown	12	12	12	12	12	12	13	12
MPN503	Unknown	10	10	10	11	10	10	12	10
MPN439	Unknown	10	10	10	10	10	10	10	10
MPN489	Unknown	10	10	10	10	10	10	10	10
MPN370	Unknown	9	11	10	9	10	9	9	9
MPN048	Unknown	10	9	9	10	9	9	10	10

**Table 5:** Functional annotation of genes with no variants

KEGG BRITE hierarchy		Count	Genes
mpn03100	Non-coding RNA	37	tRNAs, 5S rRNA, 4.5S rRNA, RNaseP RNA
mpn01000	Enzymes	28	def, deoC, dhfr, gatB, gcp, gidB, grs1, lacA, lgt, lip2, lspA, nox, pheS, polA, ppnK, pstB, rimK, rnc, udk, upp, yaaC, yacA, ygiH, yjeQ, yjfU, yjfW, MPN047, MPN479
mpn03011	Ribosome	25	50S and 30S ribosomal proteins, 5S rRNA
mpn0200	Transporters	9	amiD, permease, glnQ, dnaK, pstA, pstB, oppB, yjfU
mpn03029	Mitochondrial biogenesis	9	grs1, gatB, dnaJ, dnaK, groEL, groES, YidC, rpsP, ssb
mpn03009	Ribosome Biogenesis	5	gidB, rnc, rimK, yjeQ, rbfA, spg
mpn03016	Transfer RNA biogenesis	5	gidA, grs1, gcp, RNaseP RNA, pheS, yacA
mpn03110	Chaperones and folding catalysts	4	dnaJ, dnaK, groEL, groES, trx
mpn03036	Chromosome	4	gidA, gidB, rnc, scpB, soj
mpn02044	Secretion system	4	yidC, secE, secG, MPN680
mpn03400	DNA repair and recombination	4	polA, recA, rpoE, ssb
mpn00194	Photosynthesis proteins	3	atpG, atpF, atpE
mpn03012	Translation factors	2	infA, efp
mpn01007	Amino acid related enzymes	2	grs1, pheS
mpn03032	DNA replication	2	polA, ssb
mpn04147	Exosome	2	groEL, dnaK
mpn01002	Peptidases	2	lspA, gcp
mpn03021	Transcription machinery	1	rpoE
mpn01004	Lipid biosynthesis proteins	1	ygiH
mpn04090	Cellular antigens	1	cdd
mpn04812	Cytoskeleton proteins	1	soj

### Gene specific analysis

**Fig. 4:**
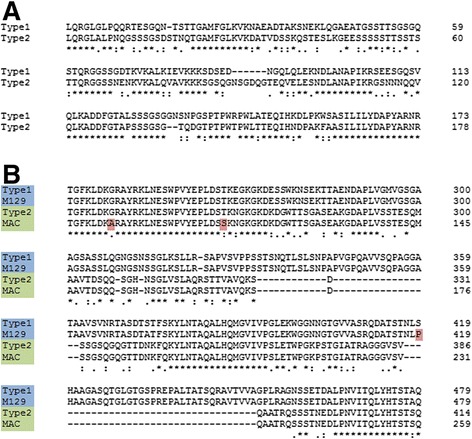
Multiple protein sequence alignments showing the differences in P1 and ORF6 between type 1 and type 2 strains. **a** The large region of variation in P1. Type1 is representative sequence for all type 1 strains and type2 is representative of all type 2 strains. **b** The large region of variation in ORF6. Type1 is representative of all type 1 strains, except M129, which is also shown (differences in M129 highlighted in red). Type2 is representative of all type 2 strains except for MAC, which is also shown (differences in MAC highlighted in red)

**Fig. 5:**
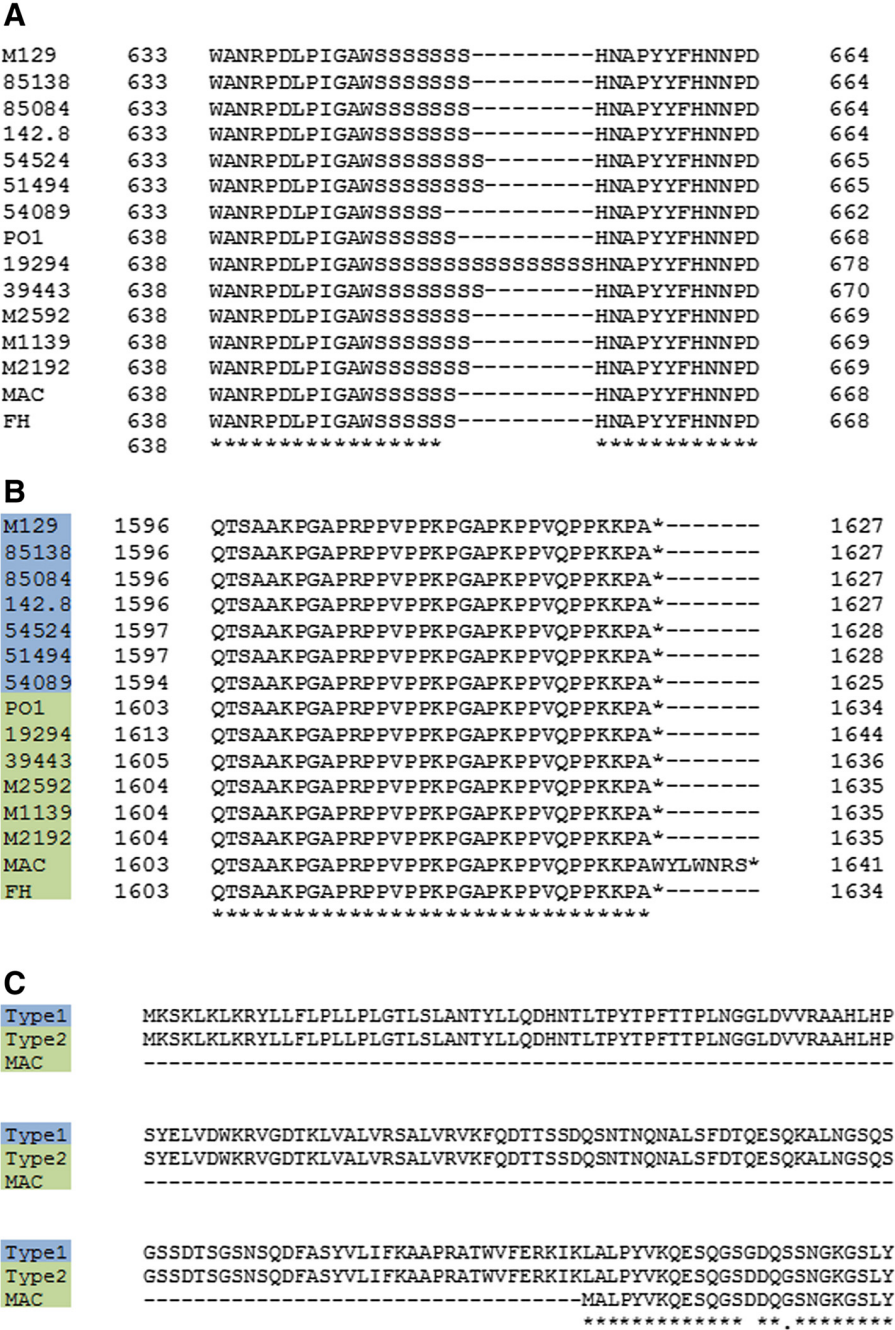
Multiple protein sequence alignments showing strain-specific differences in P1 and ORF6. Type1 and 2 strain names are highlighted in blue and green, respectively. **a** A poly-serine repeat in P1 with varying lengths in various strains. 19294 has a uniquely long allele of the poly-serine repeat, and the repeat-length in the other strains does not strictly correspond to strain type. **b** A stop-loss mutation in MAC results in an additional 7 amino acids added to the protein sequence. **c** A pair of frameshifts results in the truncation of the beginning of ORF6 in MAC. Type1 and Type2 are representative sequences for type 1 and other type 2 strains, respectively. The new protein is predicted to use an alternate start codon by RAST: the starting methionine in MAC is the same codon as that which codes for the leucine in other type 1 and type 2 strains

The two adjacent genes P1 and ORF6 code for critical components of the *M. pneumoniae* adhesin complex. These genes are within the largest region of sequence polymorphism between type 1 and type 2 strains (Fig. 1) and are the top two named genes by non-synonymous SNP count. To examine the variance in these genes further, the protein sequences were aligned and found to have identical amino acid sequences for most of their length. However, for both P1 and ORF6, there was one region where type 1 and type 2 strains had virtually no sequence identity. The alignments for the regions of high variation are shown in Fig. 4. Other variants found in these genes not in the large region of variation are listed in Additional file 3: Table S3 and Additional file 4: Table S4. Most of these other variants, like the sequence of the large regions of variation, are subtype specific, rather than strain specific. However, there were also some unique, strain specific variations with potentially significant effects in both P1 and ORF6 (Fig. 5). One of these was a variation in the number of AGT trinucleotide repeats coding for serine in a region between the repetitive elements RepMP4 and RepMP2/3 [48] within the P1 gene. The number of serine repeats ranged from 5–17 with strain 19294 bearing the longest (Fig. 5a).

MPN372 codes for the CARDS toxin, an important virulence factor of *M. pneumoniae*. There were no non-synonymous variants in any of the type 1 strains, and one non-synonymous SNP common to all of the type 2 strains (T1112G, I371S). Additionally, the FH and M2192 strains each had one unique non-synonymous SNP (FH: C74T, S25L and M2192: G1507A, G503S). The unique SNP in FH was near, but not part of, the sequences comprising the active site of the CARDS toxin.

**Fig. 6:**
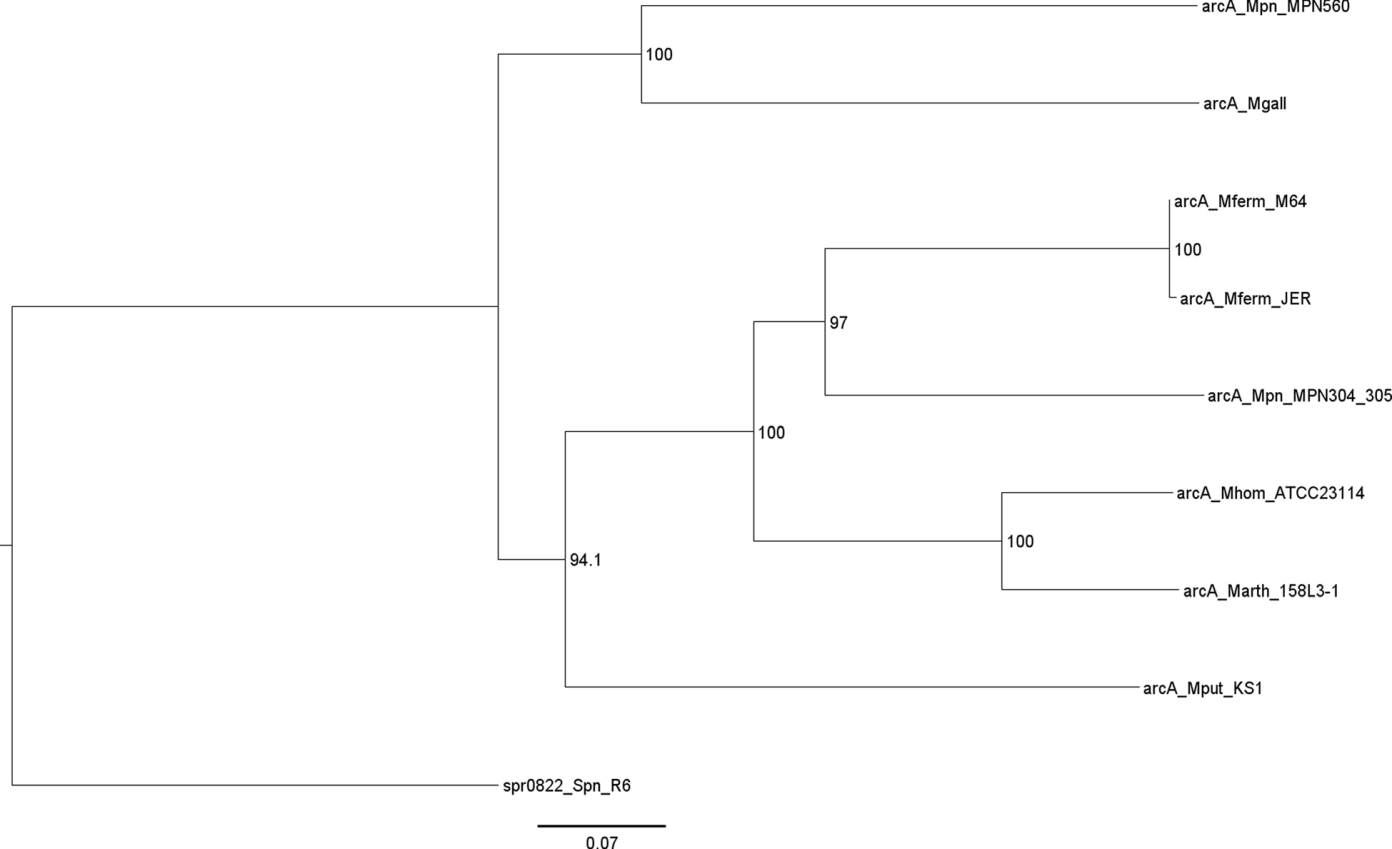
Phylogenetic tree of ArcA. The protein sequences of the two ArcA protein sequences from *M. pneumoniae* (MPN304 and MPN560), ArcA sequences from other *Mycoplasma* species, and the protein sequence of arginine deiminase from *Streptococcus pneumoniae* strain R6 (spr0822_Spn_R6) were aligned and a tree was generated from the alignment. Confidence values, represented as percent of supporting bootstrapping iterations are shown for each node. Scale, in differences per site, is indicated at the bottom

Our attention was also drawn to the *M. pneumoniae arcA* gene, an essential component of the arginine deiminase pathway that is thought to be inactive in *M. pneumoniae* [49]. There are two copies of *arcA* in *M. pneumoniae*, MPN304 and MPN560. MPN304 is truncated by a frameshift yielding a premature stop codon and is contiguous to *arcC*. MPN560 is not truncated but is found in another part of the genome. There were no variants in the coding sequence of either gene (before premature stop for MPN304) in any of the 15 sequenced strains. We also aligned the amino acid sequence of both proteins against the amino acid sequence of ArcA of several other *Mycoplasma* species. The protein sequence of arginine deiminase from *Streptococcus pneumoniae* strain R6 (spr0822) was included as an outgroup. MPN304 and MPN560 were more similar to ArcA sequences from other species than they were to each other, with MPN304 being most similar to ArcA from *Mycoplasma fermentans* and MPN560 being most similar to ArcA from *Mycoplasma gallisepticum* (Fig. 6).

The type I restriction and modification (R-M) enzymes protect bacteria from invading foreign DNA. They are composed of three types of subunits: *hsdR* encodes the restriction (R) subunit, *hsdM* encodes the modification (M) subunit and *hsdS* encodes the DNA sequence specificity (S) subunit [50]. They are pentameric enzymes with two R subunits, two M subunits and one S subunit. The S subunit is composed of two target recognition domains (TRDs) and each TRD comprises a DNA-binding domain and an alpha helical dimerization domain. The M129 genome has 2 *hsdM* (MPN198 and MPN342) and 10 *hsdS* genes scattered across the genome (Table 6). The *hsdR* gene contains frameshift mutations resulting in small ORFs (MPN345, MPN346, and MPN347) that are predicted to be nonfunctional. No prophages, conjugative elements, or genes unique to any one of the genomes were identified from the genome sequences. It is striking that the genome sequences of *M. pneumoniae* revealed so little evidence of horizontal gene transfer and the absence of an intact *hsdR*.

**Table 6:** *hsdS* genes in *M. pneumoniae* strain M129

Gene	Location (M129)	TR sequence	Amino acid repeat
MPN089	111610–112617	CCGAGCTAAGCG	AELS
MPN201	244484–245569	CCGAGCTAAG	AEL
MPN285	340244–341533	CCGAGCTAAGTG(A)	A(T)ELS
MPN289	347169–347732	CCGAGCTAAGCG	AELS
MPN290	347871–348308		
MPN343	409562–410863	CCGAACTAAGCG	AELS
MPN365	435618–436730	CCGAGCTAAGCG	AELS
MPN507	617366–618457		
MPN615	738245–739351	CCGAGCTAAGCG	AELS
MPN638	764400–765527		

**Fig. 7:**
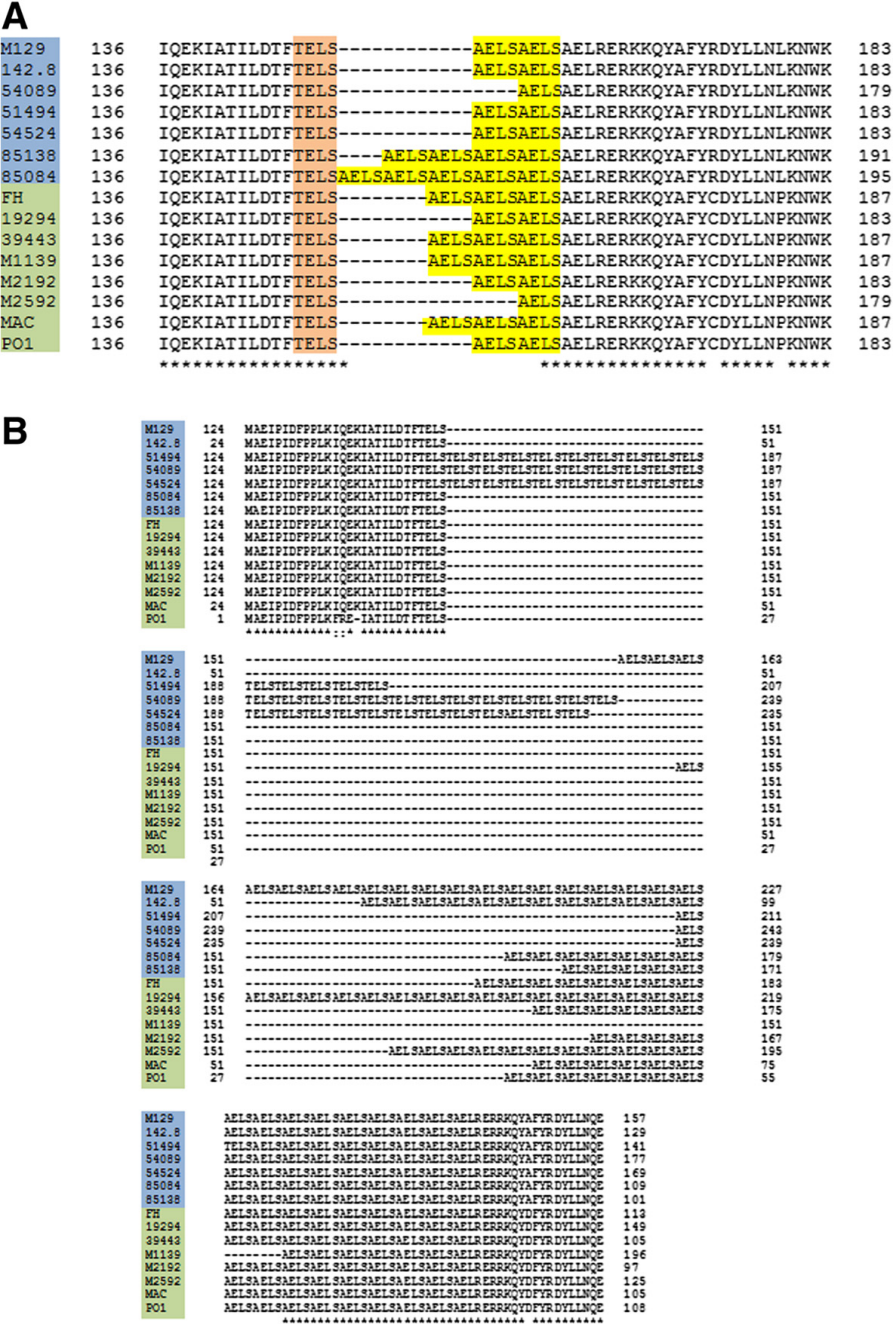
Multiple protein sequence of the variable regions in the *hsdS* genes. Both copies of the *hsdS* gene had a repetitive region of varying length consisting of TELS and AELS units (highlighted in orange and yellow, respectively). Note that in both copies, the length and composition of the repeat does not correspond to strain subtype. Strain names are to the right and highlighted in blue for type 1 and green for type 2. **a** Repeat region in the MPN089 copy of the *hsdS* gene. This is part of the variation in the 108000–126000 region shown in Fig. 2c. **b** Repeat region in the MPN343 copy of the *hsdS* gene. This is the variation in the 409700–410900 region shown in Fig. 2c

The 10 *hsdS* genes are found in all sequenced strains (Table 7). MPN289 and MPN290 appear to be two truncated subunits derived from an integral *hsdS* locus that was interrupted by a point mutation resulting in a stop codon. MPN365 and MPN615 in all sequenced type 2 strains are truncated due to a premature stop. MPN285 is also truncated in 3 strains (MAC, PO1, and 142.8) due to frameshifts. Interestingly, a 12-bp tandem repeat (TR) corresponding to a 4-amino acids repeat (AELS or TELS) within the first alpha helical dimerization domain was found in 7 out of the 10 *hsdS* genes (Table 6). The copy number of this TR varies in 6 out of the 7 *hsdS* genes among the 15 strains (Table 7). It also varies in the same strains from different passages/laboratory conditions, e.g. in published M129 and FH genomes and our resequenced M129 and FH genomes (Table 7). Because two copies of the *hsdS* gene (MPN089 and MPN343) are part of two of the strain specific genomic structure variants annotated by MAUVE, we aligned the sequences of these proteins to look at variations in these genes. In both copies of the *hsdS*, the main source of variation is the TR region of varying length with two different repetitive units (TELS and AELS). The repeat in MPN089 consists only of AELS units, although all strains have one TELS unit, like all other TR-containing *hsdS* genes (Fig. 7a). The copy number of the repeat varies from 2 to 6 and does not correspond to strain subtype. However, in MPN343, the repeats are much longer in type 1 strains (10 – 16 copies) compared to type 2 strains (1 – 2 copies). Three type 1 strains (51494, 54089, and 54524) have long repeats of mixed TELS and AELS unit (Fig. 7b and Table 7).

**Table 7:** Tandem repeat copy numbers of each *hsdS* gene in 15 *M. pneumoniae* strains

Strain		MPN089	MPN201	MPN285	MPN289	MPN290	MPN343	MPN365	MPN507	MPN615	MPN638
Type 1	M129	**5**	0	**15**	**4**	0	**16**	1	0	1	0
	M129 rs	**3**	0	**28**	**3**	0	**12**	1	0	1	0
	142.8	3	0	21	5	0	12	1	0	5	0
	51494	3	0	24	1	0	10	1	0	4	0
	54089	2	0	33	4	0	13	1	0	4	0
	54524	3	0	31	6	0	11	1	0	5	0
	85084	6	0	16	3	0	13	1	0	4	0
	85138	5	0	14	4	0	13	1	0	5	0
Type 2	FH	**5**	0	**15**	**4**	0	1	**1**	0	**1**	0
	FH rs	**4**	0	**17**	**2**	0	1	**5**	0	**4**	0
	19294	3	0	26	4	0	1	1	0	4	0
	39443	4	0	15	6	0	1	3	0	1	0
	M1139	4	0	7	2	0	1	1	0	2	0
	M2192	3	0	13	6	0	1	1	0	4	0
	M2592	2	0	20	5	0	1	1	0	3	0
	MAC	4	0	15	3	0	2	3	0	4	0
	PO1	3	0	16	6	0	1	4	0	4	0

Notes: Strain FH rs and M129 rs are our resequenced strains. Numbers in bold font indicate variations between the published and resequenced genomes

Macrolide resistance is increasing in *M. pneumoniae* and is often associated with mutations in 23S rRNA. The macrolide resistant strain 54089 was found to have a point mutation, A2063G (*E. coli* numbering), in its 23S rRNA gene. This mutation is common among macrolide resistant isolates of *M. pneumoniae* [51, 52].

## Discussion

### Quality of genome assembly

We present here 15 *M. pneumoniae* genomes in single, circular contigs. Our alignments of these genomes did not detect any apparent chromosomal alterations that were not found in other strains. Additionally, our comparison of the resequenced M129 strain with the original M129 reference sequence suggests a low level of assembly errors. Variants conserved in all sequenced strains likely represent errors in the original reference genome but some variants may be mutations that arose in the laboratory in which M129 was first sequenced. Similarly, variants found only in the resequenced M129 genome may be the result of assembly errors or mutations that occurred in our laboratory. Such variants were found in only two genes. These findings suggest that our genome assemblies are high quality and that the resequenced M129 genome is more accurate than the original.

### Comparison of the genome assemblies

Overall the similarity between the *M. pneumoniae* strains was striking with > 99 % sequence identity. The similarity within each subtype group was even stronger with less than 0.1 % difference among strains of the same subtype. The type 1 and type 2 groups of strains appear to be clonal as are some other bacteria species such as *Mycobacterium tuberculosis* [53]. Despite their geographic separation, the strains may have only recently diverged. The differences between the type 1 and 2 strains were concentrated to specific areas of the genome, rather than being evenly distributed. This suggests the existence of positive selection pressure for some variants, as might be expected for genes coding for proteins that interact with host cells such as the P1 adhesin.

We identified two large regions of genomic structural variation. These intervals were located at 557178–560601 and 713023–713984 in M129, and both were type specific. The type 2-specific insertion (Fig. 2b) contained lipoprotein genes and was identified as an insertion event previously reported only in strain 309 [54]. This block was found in all of our sequenced type 2 strains, including FH, making it a type 2 strain signature, rather than a unique feature of strain 309. The type 1 specific insertion (Fig. 2c) spanned only genes encoding hypothetical proteins.

### P1 and ORF6

The tightest clustering of the genomic differences between the type 1 and type 2 strains are found in two contiguous, functionally related genes: P1 and ORF6. Both genes are in the same operon and code for components of the *M. pneumoniae* adhesin complex, which is necessary for successful colonization of the respiratory tract [55]. The major sequence differences in the P1 and ORF6 genes between type 1 and type 2 strains are localized to specific domains of the proteins, rather than scattered across the protein. The P1 and ORF6 genes each have a long region of divergence between the type 1 and type 2 strains while the rest of the protein sequence is almost completely identical. For each gene, the region of divergence is about 350 bp, across all strains. In the case of P1, this region of variation has been mapped to a known surface-exposed domain [56]. The large regions of variation in both the P1 and ORF6 genes are within RepMP sequences. RepMP sequences are found throughout the *M. pneumoniae* genome, and previous studies suggest that recombination between RepMP sequences is responsible for antigenic variation [57, 58]. Our data support these prior studies. The within-type consistency between the type 1 and type 2 suggests that such recombination is a rare event. Our data also suggest that while type 1 and type 2 strains diverged via recombination events in the P1 and ORF6 genes (among other loci, Fig. 2), they have been relatively stable evolutionarily at these loci for at least the last six decades. This stability further suggests that the function of P1 and ORF6 is critical for survival of both type 1 and type 2 strains, even though the function of the proteins may be subtly different in each strain subtype. It is also possible that recombination events involving the RepMP sequences regularly occur but that they are immediately out-competed by the superior type 1 and type 2 variants.

Besides the variable RepMP sequences in the P1 gene, an AGT trinucleotide repeat variation was observed in all strains. This variation was previously reported in a total of 85 clinical isolates from China in two studies (repeat 5–16 times) [59, 60]. Serine repeats may form a hinge structure of a protein and hinge bending motions play an important role in catalysis and protein-ligand interactions [61]. The global P1 protein structure contains three domains that are linked by the flexible hinges [56]. The serine repeats are located in conserved domain I, close to but not included in the predicted flexible hinges. Although P1 is a surface antigen clearly related to adherence, no known protein functions such as ligand binding have been clearly predicted or characterized to it [56]. It is possible that the serine repeat variation could potentially affect its interaction with the host. Interestingly, we showed that strain 19294 has a uniquely long expansion of the polyserine repeat, and this strain has unusual morphology, as seen by electron microscopy, compared to most published photomicrographs of *M. pneumoniae* [62]. The unique expansion of the polyserine repeat may cause this phenotype by changing the way P1 folds or by changing its flexibility, and therefore its interactions with other proteins in the adhesin complex. As protein glycosylation at serine residues has recently been described in mycoplasma, the serine repeat might be a region that is heavily glycosylated with potential consequences on P1 function [63].

The functional effects of these and other variable domains in P1 and ORF6 should be the subject of future studies, but our results already suggest that a large fraction of the functional differences between type 1 and type 2 *M. pneumoniae* strains lies within one protein complex and two genes coding for components of that complex.

### CARDS toxin

The CARDS toxin is an important virulence factor of *M. pneumoniae*. First identified in 2006 as a surfactant protein A-binding protein, recombinant CARDS toxin induces ADP-ribosylation of multiple cellular proteins and vacuolization of host cells both *in vitro* and *in vivo* in rodents and primates [9, 13]. Production of CARDS toxin is upregulated by growth of *M. pneumoniae in vivo* in mammalian lung [64]. Functional analysis of the recombinant toxin reveals that the ADP-ribosylating activity resides in the N-terminal region of the protein while the cell membrane-binding and vacuolating activities are dependent on the C-terminal region [65]. Unlike P1 and ORF6, the CARDS toxin gene has very little variation among strains. There was only one SNP distinguishing the type 1 and type 2 strains. Two of the type 2 strains bear one unique SNP each, but it is not clear whether these SNPs might affect toxin function. The N-terminal S25L SNP in FH lies between two of the three conserved domains of the pertussis superfamily 1 region but does not appear to be conserved. The G503S SNP in M2192 might affect functions known to be associated with the C-terminal of the protein (the receptor binding/internalization and vacuolating activities) but the structure-function relationships underlying these activities is unknown at present [9, 65]. Additionally, we found no SNPs upstream of the gene that could potentially alter promoter function. These findings suggest that the CARDS toxin is under little selective pressure to vary.

### Arginine deiminase

Arginine deiminase activity has never been demonstrated in *M. pneumoniae*. Enzyme function is thought to be inactive due to a frameshift leading to a premature stop codon in the *arcA* gene (MPN304). We confirmed the existence of this premature stop in all of the 15 sequenced strains. However, we also observed another, intact copy of *arcA* (MPN560) in all 15 strains. The prematurely stopped copy of *arcA* was contiguous to *arcC*, while the intact copy is in a different part of the genome and appears to have a different origin based on protein sequence alignment (Fig. 6). It is likely that MPN304 was the “original” copy of *arcA* and that MPN560 was acquired later. What is most striking is that both copies of *arcA* have no nonsynonymous variants in the entirety of their coding sequences in all of the 15 sequenced strains. The lack of variation in these genes at both loci suggests that the genes are not diverging and hence may be functional. Although previous studies have suggested that the arginine deiminase pathway is inactive in *M. pneumoniae* [49], ArcA (encoded by MPN560) protein has been detected by proteogenomic assay [66]. ArcA may have a regulated activity that is not observable under prior assay conditions and/or might be involved in cellular function(s) other than arginine metabolism. All of these results warrant further study into the activity and the possible inducibility of the arginine deiminase pathway in *M. pneumoniae*.

### *hsdS* variation

Each of the strains examined had 10 copies of *hsdS* scattered throughout the genome, eight of which were identified as transcribed and 5 as translated by transcriptome and proteome analysis of M129 [1, 3, 67]. Multiple copies of *hsdS* genes are also found in other mycoplasma species, e.g., 9 in *M. suis* [68] and 21 in *M. haemofelis* [69]. *M. pulmonis* has *“*only*”* 6 *hsdS* genes but about 2 dozen *hsdS* variants, all of which are functional, can be generated by high-frequency DNA inversions with the site-specific recombination sites located within the *hsdS* coding regions [70]. *M. pneumoniae* should lack type I restriction endonuclease activity because of the apparent absence of a functional *hsdR* gene. Nevertheless, the HsdS proteins can combine with the HsdM proteins to form a functional type I modification enzyme. Indeed, the DNA methylome of *M. pneumoniae* reveals a type I DNA modification enzyme activity that recognizes the target sequence GAN_7_TAY [67]. None of the *hsdS* genes of *M. pneumoniae* should be thought of as orphans because each of the HsdS proteins can complex with any of the HsdM subunits that are available. Hence, a small number of *hsdM* genes can support a large number of *hsdS* genes to generate a system in which multiple modification enzymes with differing DNA sequence specificities are active simultaneously.

We observed a 12-bp TR within the dimerization domains in 7 *hsdS* genes, and the TR copy numbers vary in 6 of *hsdS* in all strains and in the same strain from different passages. Tandem repeats were also identified in the *hsdS* genes of *M. haemofelis* genome [69]. It is known that the length of the alpha helices determines the number of nucleotides in the non-specific spacer of the DNA recognition sequence and thus variations in this domain change the target specificity [50, 71]. Hence, the gain or loss of TR units as would be expected to occur as a result of slipped strand mispairing during DNA replication would alter the DNA methylome. The specificities of the HsdS proteins may vary during infection as has been reported for the type I restriction enzymes in *M. pulmonis* [72]. Even the truncated copies of *hsdS* may be functional as have been described for other bacteria [73]. Maintaining so many variable *hsdS* genes in the genome suggests epigenetic mechanisms for gene regulation [74].

## Conclusions

It has been estimated based primarily on rRNA analysis that the mycoplasmas are evolving more rapidly than most bacteria and that *M. pneumoniae* and closely related species are evolving more rapidly than most other mycoplasmas, suggesting a high mutation rate [75, 76]. Nevertheless, the complete sequence and assembly of 15 *M. pneumoniae* genomes from isolates collected over the past 6 decades from diverse localities across the globe demonstrate striking conservation of most genes within the two identified subtypes, including the CARDS toxin gene, an important virulence factor. Two genes coding for ArcA, a protein that is integral to the function of arginine deiminase, were also found to be completely conserved both in the apparently prematurely stopped MPN304 as well as the apparently functional MPN560. The major region of variability occurs in the P1 and ORF6 genes associated with the adhesin complex. One isolate with unique cell morphology was found to have an extended polyserine region in P1. Multiple *hsdS* genes with variable TR numbers were identified in all 15 genomes, suggesting the importance of epigenetics in this species. These data provide the basis for further studies on the evolution and structure-function pathobiology of this highly specialized pathogen.

## Additional files

Additional file 1: Table S1. Initial assembly characteristics. (XLSX 19 kb) Additional file 2: Table S2. Genes with no variants. (XLSX 12 kb) Additional file 3: Table S3. Variants in the P1 gene excluding the large variation region (nucleotides 181509-181906), relative to the M129 reference strain. (XLSX 13 kb) Additional file 4: Table S4. Variants in ORF6 excluding the large variation region (nucleotides 186572-187144), relative to the M129 reference strain. (XLSX 11 kb)
